# Biodegradable 3D-Porous Collagen Matrix (Ologen) Compared with Mitomycin C for Treatment of Primary Open-Angle Glaucoma: Results at 5 Years

**DOI:** 10.1155/2015/637537

**Published:** 2015-05-19

**Authors:** Fei Yuan, Lei Li, Xiuping Chen, Xiang Yan, Liyang Wang

**Affiliations:** ^1^Department of Ophthalmology, Zhongshan Hospital of Fudan University, Shanghai 200032, China; ^2^Department of Ophthalmology, Eye and ENT Hospital of Fudan University, Shanghai 200031, China

## Abstract

*Purpose*. To evaluate the effectiveness and safety of the Ologen as an aid for trabeculectomy performed for primary open-angle glaucoma compared with mitomycin C. *Methods*. In this prospective, randomized, parallel assignment, comparative study, 31 eyes of 21 primary open-angle glaucoma patients were allocated for trabeculectomy with the Ologen implant; another 32 eyes of 23 patients were treated with trabeculectomy augmented with mitomycin C. The patients were followed up for 5 years and evaluated for intraocular pressure, rate of success, status of the bleb, and adverse events. *Result*. The mean postoperative intraocular pressure was statistically different at 3 m, 6 m, 1 y, 3 y, and 5 y follow-up. The rates of both complete success (*P* = 0.017) and overall success (*P* = 0.031) in the Ologen group were significantly higher than those in the mitomycin C group. The difference of the bleb extent and vascularity was statistically significant in both groups. There was no significant difference in postoperative complication. *Conclusions*. Ologen provides higher rates of surgical success compared with mitomycin C for patients with primary open-angle glaucoma undergoing trabeculectomy. It may be a new, safe, simple, and effective therapeutic approach for treating primary open-angle glaucoma.

## 1. Introduction

Trabeculectomy as the standard procedure for the surgical management of glaucoma [1] is widely performed in patients with glaucoma to reduce intraocular pressure (IOP) since 1968. The reported success rates for primary trabeculectomies range from 67% to 84% [2]. It is well known that episcleral fibrosis and subconjunctival scarring are the major causes of failure of trabeculectomy [3]. Thus, inhibition of scar formation during the process of wound healing should promote greater success. In 1990, antifibrotic agents such as mitomycin C (MMC) improved the success rate and produced lower IOP when applied intraoperatively during trabeculectomy [4]. However, this was accompanied by increased adverse events such as the formation of avascular filtering blebs and corneal endothelial cell loss [5]. Accordingly, the development of methods to overcome these complications has become an important challenge.

Recently, tissue engineering has achieved great progress in creating biomedical devices for preventing scar formation by modifying the well-organized process of wound healing [6]. For instance, the Ologen (Ologen, Pro Top & Mediking Co./Ologen, Aeon Astron Europe, Netherlands), a new product that modulates wound healing following trabeculectomy, is a 3D collagen-glycosaminoglycan scaffold specifically designed to promote wound healing with minimal scarring in a wide range of ophthalmic surgeries. It has been used to create a prominent and healthy vascular bleb following trabeculectomy. Ologen should completely be degraded within 90~180 days after its implantation. The efficacy of Ologen has been demonstrated in animal models [7–9]. Previous studies [10–13] have also compared Ologen with MMC as an adjuvant for enhancing the success of trabeculectomy, but most of these studies report the short-term outcomes (less than 2 y) of surgery. Currently, there are no long-term data (5 y and over) demonstrating the successful use of Ologen as an adjuvant in trabeculectomy.

The purpose of our study was to compare the outcomes of trabeculectomy augmented with either the Ologen implant or intraoperative, low-dose MMC. We explored the hypothesis that the Ologen implant may be a viable alternative to the use of antimetabolite agents for trabeculectomy procedures and may provide a new, safe, simple, and effective therapeutic approach for treating glaucoma.

## 2. Materials and Methods

This study was a prospective, randomized, parallel assignment, comparative study undertaken in the Department of Ophthalmology of Zhongshan Hospital, Fu Dan University, China, between December 2005 and April 2006. The institutional ethics committee reviewed and approved this study. Written informed consent was obtained from each patient before surgery. This study followed the tenets of the Declaration of Helsinki. In this study, we enrolled 63 eyes of 44 patients into two groups. Thirty-one eyes were randomized to the trabeculectomy with Ologen group and 32 eyes to the MMC trabeculectomy group. Four eyes in Ologen group and five eyes in MMC group were excluded from the outcome analyses due to less than 5-year follow-up. All the patients were diagnosed with primary open-angle glaucoma by the same attending physician. The sequence of random allocation was generated by random number table by the trial statistician. Before surgery, sealed, opaque, standard sized envelopes were given to the surgeon and the same surgeon performed the surgery. After surgery, all the patients were examined by another attending physician. All the filtering blebs were evaluated and scored by two glaucoma subspecialists using the Indiana Bleb Appearance Grading Scale (IBAGS) in a masked fashion. Inclusion criteria were as follows: IOP > 21 mmHg and resistance to medical therapy and age ≧ 18 with a diagnosis of primary open-angle glaucoma. Exclusion criteria were known allergic reactions to porcine collagen, normal tension glaucoma, pregnant or breast-feeding women, and patients undergoing hemodialysis.

The Ologen collagen matrix was cylindrical shaped with 95% porous space. The pore sizes ranged from 20 to 200 *μ*m, with an average pore diameter of 140 ± 20 *μ*m. Its dimensions were 4.00 mm ± 0.3 mm (H) × 7.0 mm ± 0.5 mm (*Ø*). The matrix was obtained in a dry form, consisting of over 90% lyophilized, porcine hide collagen and less than 10% glycosaminoglycan; its density was 35.0 ± 7.0 mg/cm^3^, and PH value was 7 ± 0.5.

### 2.1. Surgical Methods

In the Ologen group, all patients underwent regular trabeculectomy. After raising a fornix-based conjunctival flap, the sclera was exposed, and a rectangular 5 × 5 mm^2^ wide and 300 *μ*m deep scleral flap was dissected at the 12-o'clock position. Then, a 2 × 3 mm sclerostomy was created, after which a peripheral iridectomy was performed. The sclera flap was closed using two relatively loose 10-0 nylon sutures. Before closing the conjunctiva, the Ologen implant was placed above the fornix-based sclera flap. Finally, the conjunctiva was closed with continuous 10-0 nylon sutures. With Ologen being in place, we believed that it was better to tie the sutures loosely to encourage aqueous flow. No sutures were required to secure the implant; as soon as it touched the sclera, it absorbed aqueous fluid and molded to the scleral tissue. The collagen matrix did not need to be presoaked or prepared in any way.

The patients allocated to the MMC group received trabeculectomy using a fornix-based surgery augmented with 0.2 mg/mL MMC (Bristol-Myers-Squibb) for 2 min. Topical tobramycin 0.3% and dexamethasone 0.1% (Tobradex, Alcon Pharmaceuticals, Fort Worth, Texas, USA) were given every 1~2 hours for 1 week and then every 6 hours till 6 weeks postoperatively, followed by tapering off. Follow-up visits were scheduled at 1 day, 7 days, 14 days, 1 month, 3 months, 6 months, 1 year, 3 years, and 5 years postoperatively. Each examination included measurement of the IOP (average of three separate readings), slit-lamp microscopy (including bleb appearance and anterior chamber inflammation), ophthalmoscopy, adverse events, and other complications. If postoperative IOP measurements were more than 21 mmHg after topical steroid withdrawal, an IOP-lowering medication was added.

The definitions used to evaluate the efficacy of both surgical techniques were as follows. Complete success was defined as an IOP ≦ 21 mmHg, with no additional glaucoma medications. Relative success was defined as an IOP ≦ 21 mmHg but with additional glaucoma medications. Failure was defined as an IOP > 21 mmHg with additional glaucoma medications. The combination of complete and relative success was labeled as overall success.

### 2.2. Statistical Analysis

Categorical variables were analyzed using the chi-square test and continuous variables were analyzed using the unpaired Student's *t*-test to detect differences between the groups. Discrete variables were presented as percentages and analyzed using Fisher's exact test. The data were analyzed using SPSS software (version 16.0, SPSS Inc., Chicago, IL, USA). Correlation was considered significant at *P* value < 0.05.

## 3. Results

The patient demographics were shown in Table 1. Baseline characteristics were similar in both groups. There were no statistically significant differences between the Ologen group and the MMC group with regard to the mean age, gender, preoperative IOP and mean duration of follow-up. The mean preoperative IOP was 43.07 ± 6.23 mmHg in the Ologen group and 41.41 ± 5.11 mmHg in the MMC group, and this difference was not statistically significant.

Table 2 demonstrated that the mean IOP decreased from 43.07 ± 6.23 mmHg to 15.11 ± 3.55 mmHg in the Ologen group and from 41.41 ± 5.11 mmHg to 19.98 ± 4.18 mmHg in the MMC group at 5 years follow-up. There was a statistically significant difference of mean IOP between both groups at 3 m, 6 m, 1 y, 3 y and 5 y follow-up.

**Figure 1:**
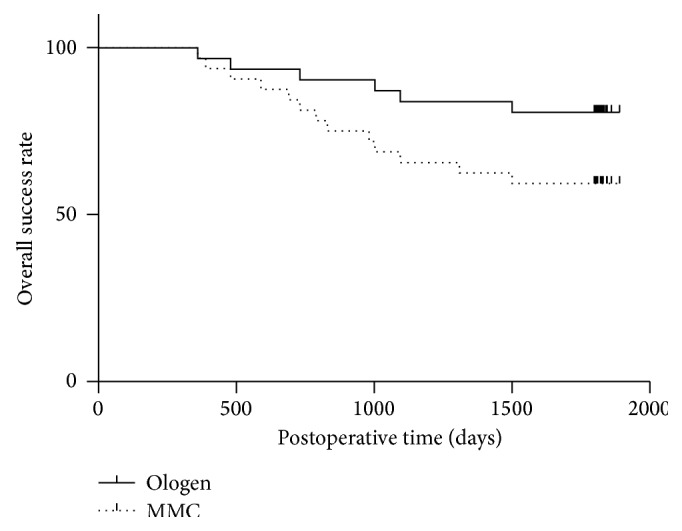
The Kaplan-Meier chart for both groups using the definition of overall success. The differences of the survival curves between both groups were statistically significant.

The rates of success and failure were described in Table 3. Another important difference between both groups was the rates of complete and overall success. At 5 years, the complete success rate was much higher in the Ologen group (61.29%) compared with the MMC group (31.25%) (*P* = 0.017). The overall success rate was 83.87% and 59.38% in the Ologen and MMC groups, respectively (*P* = 0.031). The overall success was compared using the Kaplan-Meier survival curves (Figure 1).

During the postoperative follow-up visits, we did not detect any side effects directly attributable to the Ologen implant, such as allergy or translocation of the implant.  Table 4 provided an overview of the recorded side effects. The complications included mild hyphema, hypotony, transient anterior chamber inflammation, early bleb leakage, and choroidal detachment. Using the chi-square test, the frequency of postoperative complication did not significantly differ between both groups. Hypotony was more frequent in the eyes receiving the Ologen implant compared with those receiving MMC (5 versus 4 cases, resp., *P* = 0.681). Early bleb leakage was more frequent in the MMC group than in the Ologen group (4 versus 1 eye, resp., *P* = 0.173).

**Figure 2:**
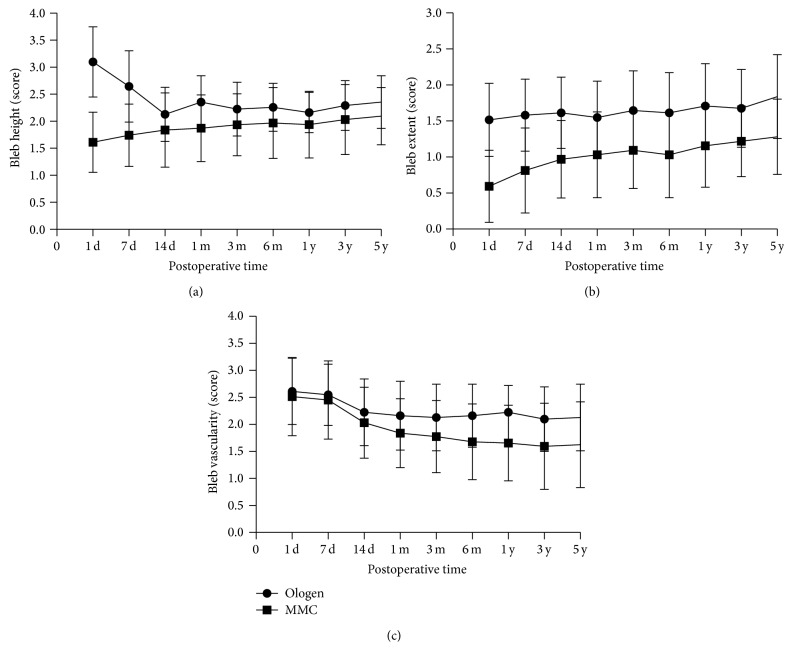
(a) The mean bleb extent during the postoperative course. The bleb extent was increased from 1 day after surgery to 5-year follow-up visit. The extent in Ologen group was larger than MMC group. (b) The mean bleb height during the postoperative course. In the early postoperative stage (up to 14 days), bleb height in Ologen group was higher than MMC group. From 14 days to 5-year visit, the bleb height was similar in both groups. (c) The mean bleb vascularity during the follow-up visit. In the early postoperative stage (up to 14 days), bleb vascularity in both groups was similar, the vascularity of MMC group was reduced from 1 month. At 5-year follow-up visit, the bleb vascularity in Ologen group was more than MMC group.

**Figure 3:**
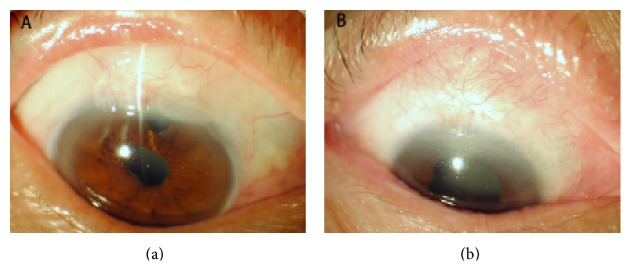
The bleb images in both groups at 5 years after surgery. (a) Elevated, diffused, well-formed filtering bleb in an eye that received Ologen implant trabeculectomy, with vascularization similar to that in the adjacent conjunctiva. (b) Flat, no function filtering bleb in an eye that received MMC trabeculectomy.

The filtering blebs were evaluated and scored by the Indiana Bleb Appearance Grading Scale (IBAGS). The parameters for the IBAGS were height, extent, and vascularity. We found that the bleb extent was increased from 1 day after surgery to the 5-year follow-up visit. There were statistically significant differences of the bleb extent between both groups during the whole follow-up visit (Table 5) (Figure 2(a)). In the early postoperative stage (up to 14 days), bleb height in Ologen group was higher than in MMC group. From 14-day to 5-year visit, the bleb height was similar in both groups (Table 5) (Figure 2(b)). All the implanted Ologens were degraded within 180 days. From 3 months, there were statistically significant differences of the vascularity between both groups (Table 5) (Figure 2(c)). At 5-year follow-up visit, the blebs in the eyes with the Ologen implants were more vascular and diffuse compared with the eyes treated with intraoperative MMC and did not show any avascular areas (Figure 3).

## 4. Discussion

Glaucoma is one of the major causes of blindness and cannot yet be cured [14]. Trabeculectomy has been used for more than 40 years and still is the most common incisional surgery for glaucoma [15]. According to several histological studies, postoperative scarring is a major problem that affects the long-term success of trabeculectomy. Since the 1980s, antimetabolite agents such as MMC, which reduce fibroblast proliferation in the subconjunctival space and in Tenon's capsule [16] and thereby inhibit scar formation, have been widely used to augment the success rates of trabeculectomy [4]. However, because of the toxicity associated with such agents, there is a greater risk of complications, such as corneal endothelial cell loss [5], cystic thin avascular bleb, choroidal detachment [17], endophthalmitis [18], and late-onset bleb leakage (>3 months after surgery) [14]. Implantation of Ologen in the subconjunctival space offers a new opportunity to prevent the above-mentioned complications associated with MMC-augmented filtering surgery and, due to its natural characteristics, to avoid early scar formation. Ologen is composed of a porous matrix of cross-linked atelocollagen and glycosaminoglycan. It contains thousands of microscopic pores and can induce fibroblast growth, leading to a well-organized and healthy healing process. In trabeculectomy surgery, Ologen provides space with a dynamic and physiological aqueous reservoir system because it is placed directly over the scleral flap and under the subconjunctival space. The implant influences the healing process by forcing the fibroblasts and myofibroblasts to grow into the pores and secrete a loose connective tissue matrix, thereby creating a mature bleb structure. Subsequently, Ologen is biodegraded by the body within 90~180 days from its implantation. Theoretically, this implant can potentially decrease scar formation and improve the surgical success of trabeculectomy without the adjunctive use of antifibrotic agents.

Recent studies in tissue engineering show that scar formation at the level of the subconjunctival space and over the scleral flap after trabeculectomy is due to the manner in which fibroblasts deposit collagen. The wound-healing response is the most important determinant of the final IOP after trabeculectomy. Excessive postoperative scarring significantly reduces the success rate. In our study, both groups demonstrated a significant reduction in the mean IOP at 5-year follow-up. Both surgeries were efficient in lowering IOP significantly from the preoperative level, as evidenced by a significantly lower IOP at all the follow-up visits in both groups. There was a statistically significant difference in the mean IOP between both groups at 3 m, 6 m, 1 y, 3 y, and 5 y follow-up. Both groups also differed significantly in the rates of complete and overall success. At the end of year 5, complete success was observed in 61.29% of the eyes in the Ologen group compared with 31.25% of the eyes in the MMC group (*P* = 0.017). The overall success rate was 83.87% and 59.38%, respectively (*P* = 0.031) (Figure 1). Our study found that the Ologen group had a significantly lower IOP and higher success rate than the MMC group. This was in contrast to the results reported by Rosentreter et al. [10], who observed a significantly greater rate of success in the MMC group in comparison to the Ologen group at 1-year follow-up. However, in their study, they evaluated only 10 cases in each group, and the follow-up time was shorter than ours.

During the follow-up visits, postoperative complications were similar in both groups. We did not detect any possible Ologen-related specific side effects, such as allergy or translocation of the implant. However, bleb leakage was very common in the early postoperative period (<7 days postoperatively) in the MMC group (4 of 32 cases) compared with the Ologen group (1 of 31 cases). At 1 week after surgery, no bleb leakage was observed in the Ologen group, whereas bleb leakage was detectable in 3 of 32 cases in the MMC group.

The long-term rate of complete success of filtering surgery depends not only on a good surgical technique but also on the steps taken to achieve a normalized bleb structure and maintain a dynamic balance between aqueous humor production and drainage, and such an approach guarantees a sufficiently preserved subconjunctival space during the process of wound healing. In our study, the filtering blebs were evaluated and scored by IBAGS which included height, extent, and vascularity of the bleb. Filtering blebs were observed in both groups, though we found several differences in the morphology of the blebs. We found that the bleb extent was increased from 1 day after surgery to the 5-year follow-up visit. There were statistically significant differences of the bleb extent between both groups during the whole follow-up visit (Table 5) (Figure 2(a)). In the early postoperative stage (up to 14 days), bleb height in Ologen group was higher than that in MMC group because of the subconjunctival implant. From 14-day to 5-year visit, the bleb height was similar in both groups due to the Ologen degradation (Table 5) (Figure 2(b)). All the implanted Ologens were degraded within 180 days. From 3 months, there were statistically significant differences of the vascularity between both groups (Table 5) (Figure 2(c)). At 5-year follow-up visit, the blebs in the eyes with the Ologen implants were more vascular and diffuse compared with the eyes treated with intraoperative MMC and did not show any avascular areas (Figure 3). The Ologen implant was designed to prevent collapse of the subconjunctival space and may also be used as an adjuvant in repairing postoperative bleb leakage [7]. These data suggested that Ologen implant in trabeculectomy can provide a healthy functional bleb.

Naveed Nilforushan et al. assigned one eye from each patient to MMC group and the other eye to Ologen group. They found that the complete success rate and the IOP level at all time points during the study were better in the MMC group. Papaconstantinou et al. [11] compared the outcomes of trabeculectomy with or without Ologen (*n* = 20 in each group) at 6-month follow-up and reported a similar success rate of 90% in both groups. Rosentreter et al. [10] and Cillino et al. [19] concluded that trabeculectomy with Ologen implantation is a safe method for trabeculectomy. Our study found that the Ologen group had a significantly lower IOP and higher success rate than the MMC group.

The meta-analysis of He et al. [20] indicated that trabeculectomy with Ologen was a safe and effective procedure in patients with glaucoma, but it did not seem to offer any significant advantages compared with trabeculectomy plus MMC due to the small number of patients and short follow-up.

In our study, Ologen was placed directly over the scleral flap and under the subconjunctival space during the trabeculectomy surgery. The procedure was very easy, and no prior preparation was required. It saved a significant amount of surgical time. Because the Ologen was not a teratogen like MMC, the nurses did not have to engage in the special handling and disposing of an antimetabolite.

Our study had several limitations. Our sample size was relatively small, and the study did not use the standardized ASOCT to evaluate the filtering bleb but merely used the slit-lamp microscopy to grade the bleb.

## 5. Conclusions

From these results, we conclude that the Ologen implant may be a new, safe, and effective alternative to MMC for improving the long-term success rate of trabeculectomy surgery and may avoid the side effects associated with the use of adjunctive therapy, such as MMC. However, larger randomized trials are required to investigate the long-term efficacy and safety of this new device. More studies investigating the wound-healing mechanisms inside the chambers of the eye, along with a longer follow-up, are under way to confirm these observations and further improve the success rate and the confidence of the scientific community in the near future.

## Figures and Tables

**Table 1:** Preoperative characteristics of patients in both groups.

Parameter	Ologen group	MMC group	*P*
	*N* = 31	*N* = 32	
Age			
Mean	55.73 ± 9.074	54.94 ± 10.525	0.522^a^
Range	33~75	31~69	
Gender			
Male/female	18/13	17/15	0.444^b^
Eye			
Right/left	13/18	15/17	0.444^b^
Mean preoperative IOP	43.07 ± 6.23	41.41 ± 5.11	0.507^a^
Mean follow-up			
Median	59.48 ± 2.74	58.81 ± 2.52	0.315^a^
Range	55~65	56~64	

^a^Independent Student's *t*-test. ^b^ *χ* ^2^ test or Fisher's exact test, as needed.

**Table 2:** Postoperative IOP comparison between the Ologen group and the MMC group.

IOP (mmHg)	Ologen group	MMC group	*P*
	(*N* = 31)	(*N* = 32)	
Preop	43.07 ± 6.23	41.41 ± 5.11	0.507
1 d postoperatively	8.90 ± 2.13	9.83 ± 2.28	0.106
7 d postoperatively	12.19 ± 2.73	11.23 ± 2.48	0.155
14 d postoperatively	13.13 ± 2.48	14.14 ± 3.22	0.177
1 m postoperatively	12.89 ± 2.41	14.05 ± 4.04	0.181
3 m postoperatively	13.07 ± 2.09	16.53 ± 4.21	0.005^*∗*^
6 m postoperatively	14.04 ± 3.07	16.81 ± 4.96	0.011^*∗*^
1 y postoperatively	14.23 ± 3.13	17.25 ± 4.38	0.003^*∗*^
3 y postoperatively	14.56 ± 3.12	18.08 ± 4.07	0.000^*∗*^
5 y postoperatively	15.11 ± 3.55	19.98 ± 4.18	0.000^*∗*^

Independent Student's *t*-test. ^*∗*^ *P* < 0.05.

**Table 3:** The success rate comparison between the Ologen and MMC groups.

Success rate	Ologen group	MMC group	*P*
	(*N* = 31)	(*N* = 32)	
Complete success	19 (61.29)	10 (31.25)	0.017^*∗*^
Relative success	6 (19.35)	9 (28.13)	0.414
Overall success	26 (83.87)	19 (59.38)	0.031^*∗*^
Failure	6 (19.35)	13 (40.63)	0.066

χ^2^-test. ^*∗*^ *P* < 0.05.

**Table 4:** Comparison of complications between the Ologen group and MMC group.

Complication	Ologen group		MMC group		*P*
	(*N* = 31)		(*N* = 32)		
	*n*	%	*n*	%	
Mild hyphema	2	6.45	3	9.38	0.668
Hypotony	5	16.13	4	12.5	0.681
Transient anterior chamber inflammation	2	6.45	3	9.38	0.668
Early bleb leakage (<7 days)	1	3.23	4	12.5	0.173
Choroidal detachment	2	9.68	1	12.5	0.535

*χ* ^2^-test.

**Table 5:** The *P* value and *T* value between the Ologen group and MMC group.

	*P* (*T*)		
	Height	Extent	Vascularity
1 d	0.000 (9.679)	0.000 (7.270)	0.506 (0.669)
7 d	0.000 (5.402)	0.000 (5.546)	0.499 (0.680)
14 d	0.106 (1.642)	0.000 (4.941)	0.227 (1.221)
1 m	0.003 (3.123)	0.004 (3.712)	0.051 (1.992)
3 m	0.068 (1.858)	0.000 (4.049)	0.035 (2.158)
6 m	0.078 (1.791)	0.002 (3.999)	0.005 (2.933)
1 y	0.089 (1.730)	0.000 (3.778)	0.000 (3.710)
3 y	0.073 (1.852)	0.000 (3.527)	0.006 (2.826)
5 y	0.046 (2.035)	0.000 (3.999)	0.007 (2.806)

Unpaired *t*-test.
